# Oxidative Stress, Advanced Glycation End Products (AGEs), and Neurodegeneration in Alzheimer’s Disease: A Metabolic Perspective

**DOI:** 10.3390/antiox14091044

**Published:** 2025-08-25

**Authors:** Virginia Boccardi, Francesca Mancinetti, Patrizia Mecocci

**Affiliations:** 1 Division of Gerontology and Geriatrics, Department of Medicine and Surgery, University of Perugia, 06120 Perugia, Italy; virginia.boccardi@unipg.it (V.B.); francesca_manci@hotmail.it (F.M.); 2 Division of Clinical Geriatrics, Department of Neurobiology, Care Sciences and Society, Karolinska Institutet, 17177 Stockholm, Sweden

**Keywords:** aging, inflammation, insulin, metabolism, neurodegeneration, oxidative stress

## Abstract

Neurodegenerative diseases such as Alzheimer’s disease (AD) are closely linked to oxidative stress and advanced glycation end products (AGEs), two interrelated processes that exacerbate neuronal damage through mitochondrial dysfunction, protein aggregation, and chronic inflammation. This narrative review explores the metabolic interplay between reactive oxygen species (ROS) and AGEs, with a focus on the AGE-RAGE (receptor for advanced glycation end products) signaling axis as a driver of neurodegeneration. Evidence from preclinical and clinical studies highlights their combined role in disease progression and underscores potential therapeutic targets. Strategies including mitochondria-targeted antioxidants, AGE inhibitors, RAGE antagonists, and metabolic interventions are discussed, along with future directions for biomarker development and personalized treatments. This review integrates current molecular insights into a unified metabolic–inflammatory model of AD, highlighting translational therapeutic opportunities.

## 1. Introduction

Neurodegenerative diseases, such as Alzheimer’s disease (AD), represent a growing global health burden, particularly in the context of rapidly aging populations. AD is the leading cause of dementia, accounting for 60–80% of all cases [1]. Dementia is a clinical syndrome marked by progressive cognitive decline that interferes with daily functioning, and AD progresses along a continuum, from preclinical stages to mild cognitive impairment (MCI) and overt dementia [2]. Two principal subtypes of AD are distinguished by the age of onset: early-onset AD (EOAD), which typically occurs before age 65 and is often linked to autosomal dominant mutations (*APP*, *PSEN1*, *PSEN2*) [3], and late-onset AD (LOAD), which manifests after age 65 and comprises most cases [4]. Unlike EOAD, LOAD is multifactorial, arising from a complex interplay of genetic predisposition (e.g., *APOE* ε4), environmental exposures, metabolic disturbances, and aging-related mechanisms. Importantly, LOAD may be considered a geriatric syndrome, reflecting its heterogeneity, multifactorial pathogenesis, and its primary risk factor, advanced age [5] (Table 1).

Over the years, several non-exclusive hypotheses have been proposed to explain AD pathophysiology [7]. The amyloid cascade hypothesis suggests that the extracellular aggregation of amyloid-β (Aβ) initiates a cascade of neurotoxic events, while the tau hypothesis emphasizes the role of hyperphosphorylated tau in neuronal dysfunction. More recently, attention has shifted to broader systemic contributors [8]. The mitochondrial cascade hypothesis posits that age-related mitochondrial dysfunction is an early event promoting both Aβ and tau pathology. Parallel theories highlight the role of neuroinflammation and metabolic dysregulation, both of which intersect at the level of oxidative stress [9]. The excessive production of reactive oxygen (ROS) and nitrogen species leads to cumulative damage to neuronal lipids, proteins, and DNA, particularly deleterious in neurons, given their high energy demand and limited regenerative capacity [9,10].

Within this oxidative and inflammatory milieu, advanced glycation end products (AGEs) have emerged as key mediators [11]. Formed through non-enzymatic glycation and oxidation of proteins and lipids, AGEs accumulate with age and are elevated in AD brains [12]. They promote the protein cross-linking and structural alterations of Aβ and tau, enhancing their aggregation and neurotoxicity. Moreover, AGEs activate the receptor for AGEs (RAGE), initiating a feed-forward loop of ROS generation, NF-κB-mediated inflammation, and neuronal apoptosis. Mounting evidence suggests that AGE–RAGE signaling acts not only as a marker, but as an upstream amplifier of the metabolic–inflammatory axis driving LOAD [13,14,15]. This places AGEs at the intersection of systemic metabolic dysfunction (e.g., insulin resistance), neuroinflammation, and classical neuropathology.

This review aims to critically examine the metabolic dimension of Alzheimer’s disease, with a particular emphasis on the interplay between oxidative stress and AGEs in the pathogenesis of LOAD. We sought to achieve the following: (1) elucidate the molecular and cellular mechanisms linking AGEs and oxidative stress to neurodegeneration, (2) highlight AGE–RAGE signaling as a central node in aging-related metabolic and inflammatory dysfunction, and (3) discuss emerging therapeutic strategies targeting AGE formation, detoxification, and receptor-mediated signaling in the context of AD.

## 2. Methodology

A comprehensive literature search was performed across PubMed/MEDLINE, Scopus, and Web of Science databases for studies published between January 2000 and May 2025, using combinations of the following keywords and MeSH terms: “Alzheimer’s disease,” “late-onset Alzheimer’s,” “oxidative stress,” “advanced glycation end products,” “AGEs,” “RAGE,” “neuroinflammation,” “mitochondrial dysfunction,” and “neurodegeneration.” We included original research articles (preclinical, clinical, and translational), systematic reviews, and meta-analyses published in English that explored mechanistic links between oxidative stress, AGEs, and Alzheimer’s pathology, while excluding case reports, editorials, and studies not directly addressing AD. Two reviewers independently screened titles, abstracts, and full texts to assess eligibility, resolving discrepancies through consensus. Data extraction focused on AGE formation, AGE–RAGE signaling, oxidative stress pathways, and therapeutic implications. Due to heterogeneity in methodologies and outcomes, the findings were synthesized narratively and organized thematically rather than through meta-analytic techniques.

## 3. Oxidative Stress and Neurodegeneration in Alzheimer’s Disease

Oxidative stress refers to an imbalance between the generation of ROS and the capacity of antioxidant systems to neutralize them, resulting in molecular and cellular damage [16]. ROS include both free radicals—such as superoxide anion (O_2_^−^·) and hydroxyl radical (·OH)—and non-radical species like hydrogen peroxide (H_2_O_2_). These are primarily generated as the by-products of mitochondrial oxidative phosphorylation and through enzymatic pathways involving nicotinamide adenine dinucleotide phosphate (NADPH) oxidases, xanthine oxidase, and cytochrome P450 isoforms [17]. Although the physiologic levels of ROS contribute to redox signaling and immune defence, their excessive accumulation compromises cellular homeostasis.

As summarized in Table 2, ROS target multiple macromolecules. In proteins, oxidation alters amino acids and structure, reducing function; in lipids, peroxidation disrupts membranes; in carbohydrates, glucose autoxidation generates reactive intermediates; and in DNA, oxidative lesions alter gene integrity and expression [18]. Over time, such injuries can evolve into various pathological conditions, including neurodegenerative disorders [19].

Oxidative damage is a pervasive and early event in the pathogenesis of neurodegenerative diseases, including AD. The central nervous system (CNS) is particularly vulnerable to oxidative insults due to its high oxygen consumption, abundance of polyunsaturated lipids, elevated metabolic rate, and limited antioxidant buffering capacity [20]. Endogenous antioxidant defences include enzymatic systems—such as superoxide dismutase (SOD), catalase, glutathione peroxidase (GPx), and peroxiredoxins—as well as non-enzymatic agents like reduced glutathione (GSH), vitamins C and E, and coenzyme Q10. These systems collectively preserve redox equilibrium and protect neuronal macromolecules from oxidative damage [17,19]. Excessive ROS promote a cascade of neurotoxic events, starting with lipid peroxidation, which compromises neuronal membrane fluidity and function. Reactive aldehyde by-products, notably 4-hydroxynonenal (4-HNE) and malondialdehyde (MDA), impair ion homeostasis, membrane-bound receptors, and synaptic signaling. ROS also oxidatively modify proteins, inducing carbonylation, nitration, and disulfide cross-linking [21]. These changes disrupt protein folding, enzymatic activity, and accelerate aggregation. The oxidative modifications of tau and Aβ enhance their neurotoxicity and propensity to form pathological inclusions, contributing to synaptic and neuronal loss [22]. Mitochondria are both a source and target of oxidative stress. ROS-induced damage to mitochondrial DNA (mtDNA)—which lacks protective histones and robust repair mechanisms—leads to impaired ATP synthesis, increased mitochondrial permeability, and further ROS production. This vicious cycle culminates in bioenergetic failure and apoptotic cell death, the hallmark features of Alzheimer’s pathology [22,23].

Postmortem analyses consistently show elevated markers of lipid peroxidation (e.g., malondialdehyde [MDA], 4-hydroxynonenal [4-HNE]), protein oxidation (e.g., carbonylated proteins), and oxidative DNA damage (e.g., 8-hydroxy-2′-deoxyguanosine [8-OHdG]) in the hippocampus and cortex of AD brains [24,25,26]. Amyloid-β (Aβ) aggregates interact with redox-active metals such as Cu^2+^ and Fe^2+^, catalyzing ROS formation via Fenton-like reactions, which amplify lipid peroxidation and membrane disruption [25]. This oxidative environment promotes mitochondrial dysfunction—characterized by mtDNA damage, respiratory chain complex impairment, reduced ATP synthesis, and further ROS production—thus creating a self-perpetuating cycle [27]. Tau protein is similarly affected, with oxidative modifications (nitration, carbonylation) altering its microtubule-binding properties, promoting hyperphosphorylation, and enhancing aggregation into neurofibrillary tangles [28]. Redox-sensitive transcription factors such as NF-κB and AP-1 become activated in microglia and astrocytes, leading to the sustained production of pro-inflammatory cytokines (IL-1β, TNF-α, IL-6) [28]. Biomarkers, including MDA, 4-HNE, protein carbonyls, 3-nitrotyrosine, and 8-OHdG, not only correlate with AD severity, but are also elevated in plasma and cerebrospinal fluid in mild cognitive impairment, suggesting a role as early indicators [24,25,26]. Notably, antioxidant defences—such as glutathione, superoxide dismutase, catalase, and glutathione peroxidase—are often diminished in AD, further compromising neuronal resilience to oxidative stress [29].

## 4. Advanced Glycation End Products, Oxidative Stress, and Neurodegeneration in Alzheimer’s Disease

AGEs are a heterogeneous group of stable, irreversible compounds formed through the non-enzymatic Maillard reaction between reducing sugars and amino groups of proteins, lipids, or nucleic acids [30]. The Maillard reaction—particularly during thermal processing—produces the end products responsible for the flavors, aromas, and savory qualities of sugar- and protein-rich foods. The evolving Western diet has markedly increased the intake of ultra-processed foods (UPFs), often enhanced with additives that, when heated, promote AGE formation. These dietary patterns are characterized by high levels of sugars and fats, the widespread use of protein supplements, and sedentary behavior, all of which have been linked to increased non-communicable disease risk. In the United States, UPFs contribute approximately 55% of the daily caloric intake in adults and nearly 62% in children [31], with similar proportions reported in the United Kingdom and other European countries [32]. High-temperature dry cooking methods (grilling, roasting, baking) and industrial processing markedly increase AGE content. Although only 10–30% of ingested AGEs are absorbed in the gastrointestinal tract [33], cohort studies show that a higher dietary AGE intake is associated with faster cognitive decline and greater dementia risk [34]. In turn, randomized controlled trials show that low-AGE diets, such as low-fat vegan regimens, can reduce daily AGE intake by over 73% compared to conventional diets, improving body weight and cardiometabolic parameters [35].

Additionally, AGEs may also form via oxidative mechanisms, particularly when ROS interact with intermediate metabolic compounds. These oxidative modifications can parallel reactions in which lipid peroxidation by-products bind to protein amino groups, giving rise to structurally analogous compounds known as advanced lipoxidation end products (ALEs) [36]. AGEs and oxidative stress exist in a mutually reinforcing loop. Oxidative conditions accelerate AGE formation, while AGEs themselves enhance ROS production through activation of RAGE. Elevated concentrations of AGEs, together with heightened expression of their primary receptor, RAGE, within tissues, can trigger and intensify various pathological processes. This occurs through the upregulation of gene transcription linked to proinflammatory cytokines and signaling molecules, ultimately leading to dysregulated protein synthesis and sustained inflammatory responses [37]. RAGE is expressed on neurons, microglia, astrocytes, and endothelial cells. Its activation triggers intracellular signaling cascades—including NADPH oxidase activation, mitochondrial ROS generation, and NF-κB translocation—culminating in a chronic pro-inflammatory and pro-oxidative state [38]. This feed-forward mechanism contributes to sustained redox imbalance, glial activation, and neuronal apoptosis, all of which are observed in the Alzheimer’s brain. Moreover, AGEs can modify critical proteins such as Aβ and tau, enhancing their aggregation and neurotoxicity. AGE-modified tau shows increased resistance to degradation and higher propensity for misfolding and fibril formation, promoting the formation of neurofibrillary tangles [39].

**Figure 1:**
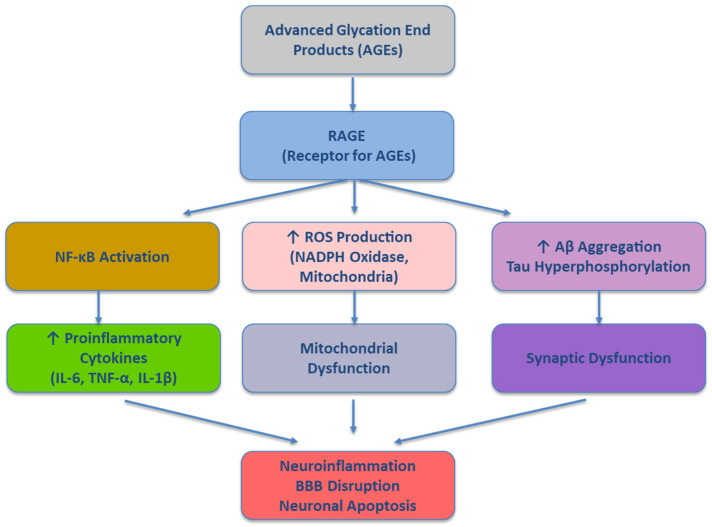
Schematic representation of the AGE–RAGE signaling pathway in Alzheimer’s disease. AGEs—advanced glycation end products; RAGE—receptor for advanced glycation end products; ROS—reactive oxygen species; NF-κB—nuclear factor kappa-light-chain-enhancer of activated B cells; IL—interleukin; TNF-α—tumor necrosis factor-alpha; Aβ—amyloid-beta; Tau—microtubule-associated protein tau; BBB—blood–brain barrier. Up arrow indicating an increase.

The AGE–RAGE interaction acts as a central node linking oxidative stress and inflammation in AD [37,39]. Specifically, the AGE–RAGE axis contributes to the increased amyloidogenic processing of APP, Tau hyperphosphorylation, mitochondrial dysfunction, autophagic impairment and cerebrovascular injury, and blood–brain barrier (BBB) compromise. Specifically, the binding of AGEs to RAGE upregulates β-secretase (BACE1) activity, leading to an enhanced production of amyloid-β (Aβ) [40]. Concurrently, the RAGE-mediated dysfunction of microglial phagocytosis impairs Aβ clearance, fostering extracellular plaque accumulation. The activation of glycogen synthase kinase-3β (GSK-3β) and mitogen-activated protein kinases (MAPKs) downstream of RAGE promotes the aberrant phosphorylation of tau proteins [41]. Moreover, AGE–RAGE signaling disrupts mitochondrial dynamics and bioenergetics, leading to elevated ROS production [42]. Again, autophagic flux is a crucial mechanism for degrading misfolded proteins and damaged organelles. The inhibition of autophagy, observed in both animal models and post-mortem AD brain tissue, leads to the intracellular accumulation of amyloid-β and hyperphosphorylated tau, thereby amplifying synaptic toxicity and neuronal injury [43]. The dysfunction of lysosomes and autophagosomes reduces the neuronal capacity to maintain protein homeostasis (proteostasis) and to remove damaged mitochondria, resulting in further oxidative stress and ROS production [43]. This creates a vicious cycle in which defective autophagy fuels neurodegeneration, making the targeted activation of autophagic pathways a potential therapeutic approach in AD. The RAGE expression on endothelial cells mediates vascular inflammation and promotes leukocyte adhesion and transmigration [44]. This activity, coupled with matrix metalloproteinase activation, facilitates BBB breakdown, which exacerbates neuroinflammation and facilitates peripheral immune cell infiltration into the CNS [45]. Animal models fed high-AGE diets or given methylglyoxal demonstrate hippocampal AGE deposition, mitochondrial dysfunction, increased amyloid-β accumulation, tau hyperphosphorylation, gliosis, and impaired memory, supporting strong biological plausibility [34]. In humans, elevated AGEs have been detected in brain tissue, cerebrospinal fluid, and the plasma of individuals with Alzheimer’s disease [46], where higher circulating AGE levels predict a faster cognitive decline, even among non-diabetic individuals [34]. Endogenous AGE production is accelerated by chronic hyperglycemia, oxidative/dicarbonyl stress, and impaired renal clearance; accordingly, populations with type 2 diabetes or chronic kidney disease consistently show elevated AGEs correlating with vascular injury markers [47]. Collectively, the AGE–RAGE signaling cascade involves multiple pro-oxidant and pro-inflammatory nodes that contribute to the neurodegenerative processes characteristic of Alzheimer’s disease. A schematic overview of this molecular pathway is presented in Figure 1. The binding of AGEs to RAGE activates NADPH oxidase and disrupts mitochondrial function, generating ROS that drive NF-κB translocation and the release of proinflammatory cytokines (IL-1β, TNF-α, IL-6). Normally, IL-4 and IL-10 restrain this response by modulating microglia, but in Alzheimer’s disease, their reduced levels in serum, cerebrospinal fluid, and brain tissue remove this anti-inflammatory brake, sustaining a pro-oxidative, pro-inflammatory state [48]. AGE–RAGE signaling also promotes tau hyperphosphorylation, amyloid-β aggregation, BBB disruption, synaptic dysfunction, and neuronal apoptosis.

## 5. The AGE–RAGE Axis as a Central Node in Aging-Related Metabolic and Inflammatory Dysfunction

**Figure 2:**
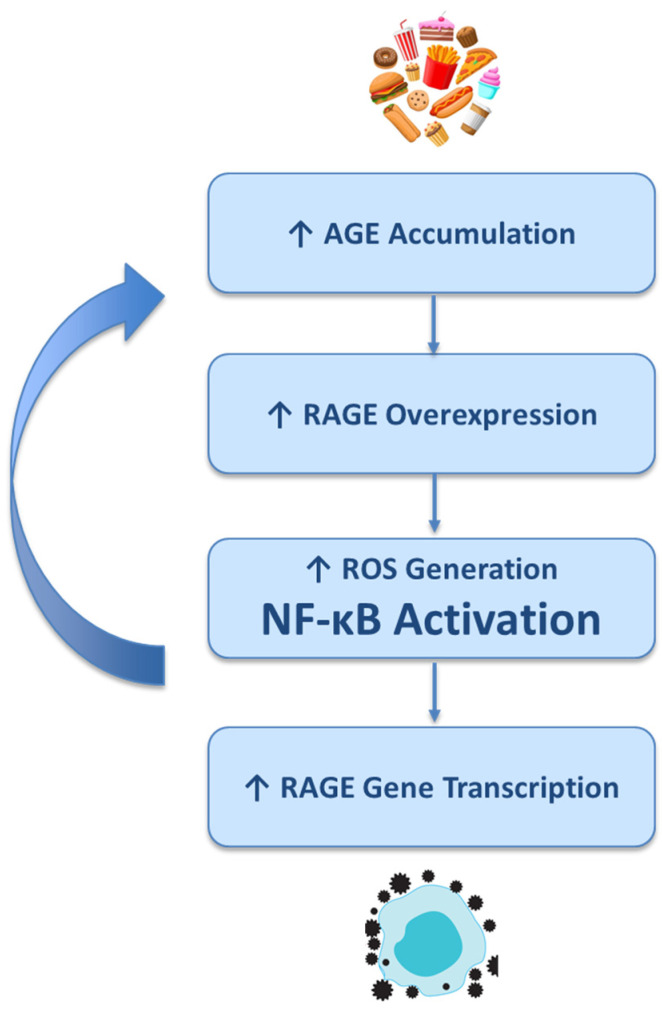
Feed-forward loop in AGE–RAGE signaling. AGE—advanced glycation end product; RAGE—receptor for advanced glycation end product; ROS—reactive oxygen species; NF-κB—nuclear factor kappa-light-chain enhancer of activated B cells. Up arrow indicating an increase.

Beyond the brain-specific effects, AGE–RAGE signaling exerts systemic metabolic and inflammatory actions that converge on the aging process itself. The interaction between AGEs and their main receptor, RAGE, constitutes a central mechanism linking oxidative stress, chronic inflammation, and metabolic dysregulation in aging and age-related diseases. RAGE is a multiligand pattern recognition receptor belonging to the immunoglobulin superfamily, and is expressed in neurons, microglia, astrocytes, endothelial cells, and peripheral immune cells [49,50]. While minimally expressed under physiological conditions, RAGE is markedly upregulated in aging tissues, particularly in response to increased AGE accumulation [51]. Upon ligand binding, the AGE–RAGE interaction initiates intracellular signaling cascades that lead to the activation of nuclear factor-κB (NF-κB), mitogen-activated protein kinases (MAPKs), NADPH oxidase, and downstream proinflammatory transcriptional programs. This results in the production of ROS, cytokines (e.g., IL-6, TNF-α, IL-1β), and adhesion molecules (e.g., ICAM-1, VCAM-1), which together foster a sustained state of inflammaging, a chronic, low-grade inflammation characteristic of biological aging [49,51]. The AGE–RAGE axis also plays a pivotal role in systemic metabolic dysfunction. AGEs impair insulin signaling and pancreatic β-cell viability, contributing to insulin resistance and type 2 diabetes, two well-established risk factors for LOAD [52]. RAGE activation in adipose tissue and skeletal muscle disrupts glucose uptake and mitochondrial function, amplifying oxidative stress and impairing cellular metabolism. In the brain, insulin resistance and impaired glucose metabolism have been observed in early AD, often referred to as “type 3 diabetes” [53]. AGE–RAGE signaling exacerbates these defects by interfering with insulin receptor substrate phosphorylation and promoting inflammatory cytokine release that inhibits insulin signaling pathways. This positions the AGE–RAGE axis as a mechanistic bridge between systemic metabolic impairment and central neurodegeneration. In the CNS, AGE–RAGE signaling promotes neuroinflammation through sustained microglial and astrocytic activation [54,55]. Microglia exposed to AGEs adopt a proinflammatory M1 phenotype, releasing ROS and cytokines that damage synapses and neurons. Astrocytes respond by downregulating neurotrophic support and enhancing glutamate excitotoxicity. These glial responses further compromise neuronal viability and synaptic plasticity [55,56]. Moreover, RAGE expression on cerebral endothelial cells contributes to BBB breakdown, allowing for peripheral inflammatory mediators and circulating AGEs to enter the brain parenchyma [57]. BBB disruption is a key early event in AD pathogenesis and facilitates further Aβ deposition, tau pathology, and metabolic toxicity. Crucially, AGE–RAGE signaling operates within a feed-forward loop, as shown in Figure 2. Increased accumulation of AGEs leads to the overexpression of their receptor, RAGE. This upregulation facilitates the generation of ROS and the activation of the pro-inflammatory transcription factor NF-κB. In turn, NF-κB enhances the transcription of the RAGE gene itself, establishing a self-perpetuating cycle. This vicious loop sustains chronic inflammation and oxidative stress, which, in turn, reinforce peripheral metabolic dysfunction (such as insulin resistance and endothelial impairment) and exacerbate central neurodegenerative cascades, contributing to the progression of age-related diseases. This dynamic creates a pathophysiological continuum in which AGE–RAGE signaling not only reflects cumulative metabolic damage but actively drives the transition from normal aging to pathological aging, including the onset and progression of LOAD.

## 6. Therapeutic Strategies Targeting AGE Formation, Detoxification, and RAGE Signaling in AD

Given the central role of AGEs and AGE–RAGE signaling in AD pathophysiology—particularly in LOAD—several pharmacological and nutraceutical strategies have been proposed to mitigate their deleterious effects. These approaches target various stages of the AGE cascade, from preventing AGE formation and promoting their detoxification to inhibiting receptor-mediated downstream signaling (Table 3).

### 6.1. Inhibitors of AGE Formation and AGE Cross-Link Breakers

AGE formation can be attenuated by agents that interfere with early steps of the Maillard reaction or that scavenge reactive carbonyl intermediates. Aminoguanidine, one of the earliest AGE inhibitors, traps reactive carbonyl species and prevents the cross-linking of proteins. While preclinical studies showed neuroprotective and anti-inflammatory effects, its clinical development was halted due to adverse events (e.g., liver toxicity) [65]. Pyridoxamine, a form of vitamin B6, acts as a carbonyl scavenger and metal chelator, showing promising results in diabetic and nephropathic models [66]. Although limited data exist in AD, its dual antioxidant and anti-glycation properties offer theoretical potential. ALT-711 (Alagebrium), a cross-link breaker that reverses AGE-related protein modifications, showed efficacy in improving vascular compliance in cardiovascular disease [66] and has been proposed for investigation in neurodegenerative contexts.

### 6.2. Enhancing AGE Detoxification and Clearance

The human body possesses endogenous detoxification systems for AGE intermediates, notably the glyoxalase pathway, which degrades reactive dicarbonyls such as methylglyoxal. Regarding glyoxalase-1 (GLO1) activation, the experimental upregulation of GLO1 in mouse models has been shown to reduce AGE accumulation, oxidative stress, and improve cognitive outcomes [60]. Agents that enhance GLO1 activity (e.g., sulforaphane, resveratrol) are under investigation for their neuroprotective potential. Glutathione, a key intracellular antioxidant, supports AGE detoxification indirectly by reducing dicarbonyl stress. GSH depletion has been documented in AD brains, suggesting that GSH replenishment (via precursors such as N-acetylcysteine or dietary interventions) could modulate AGE load and redox balance [67].

### 6.3. RAGE Antagonists and Receptor-Targeted Therapies

A major focus of drug development has been the inhibition of AGE–RAGE interaction and its intracellular consequences. Azeliragon (TTP488), an oral RAGE antagonist, reached phase II/III clinical trials for mild Alzheimer’s disease. Although early trials indicated potential benefits in reducing cognitive decline and inflammation, subsequent phase III results were inconclusive, prompting ongoing interest in better-targeted or combinatorial approaches [68]. FPS-ZM1, a high-affinity RAGE inhibitor, successfully crosses the blood–brain barrier and has shown promise in preclinical AD models. It reduces Aβ influx into the brain, suppresses RAGE-mediated inflammatory signaling, and preserves synaptic function. Further studies are needed to translate these findings into clinical applications [69]. sRAGE (soluble RAGE) functions as a decoy receptor, binding circulating AGEs and preventing them from activating membrane-bound RAGE. Reduced sRAGE levels have been reported in patients with AD, and strategies to boost endogenous sRAGE or administer recombinant forms are being explored [70].

### 6.4. Nutritional and Lifestyle Interventions

Dietary AGEs, derived from high-temperature cooking (grilled, fried, roasted foods), significantly contribute to systemic AGE burden [63]. Clinical and observational studies suggest that low-AGE diets, rich in raw or steamed vegetables, whole grains, and low-glycemic foods, may reduce circulating AGEs and improve the markers of inflammation and cognition in older adults [71,72]. Polyphenols, such as curcumin, quercetin, and resveratrol, exhibit dual activity as antioxidants and glycation inhibitors [73]. These compounds attenuate AGE formation, modulate RAGE expression, and suppress downstream inflammatory signaling [74]. While their bioavailability and pharmacokinetics remain a challenge, formulations with enhanced absorption (e.g., liposomal curcumin) are under investigation [75]. Physical exercise, by improving mitochondrial function, insulin sensitivity, and antioxidant capacity, may also reduce AGE accumulation indirectly, highlighting the importance of multimodal lifestyle interventions in modulating the AGE–RAGE axis in AD [76,77].

## 7. Conclusions

Despite encouraging preclinical evidence, the clinical translation of therapies targeting AGEs in AD remains limited. Several critical challenges impede progress, including the following: (i) the structural and functional heterogeneity of AGEs, which complicates the development of selective interventions; (ii) the absence of reliable biomarkers to quantify AGE burden and RAGE (receptor for AGEs) signaling activity within the central nervous system; and (iii) the need for early therapeutic intervention, given that AGE–RAGE-mediated damage likely precedes clinical onset by decades. Nonetheless, innovative strategies such as nanocarrier-based drug delivery systems, gene therapies aimed at modulating RAGE expression, and combinatorial approaches that simultaneously target metabolic, inflammatory, and oxidative stress pathways are emerging as promising avenues to overcome current limitations. The therapeutic disruption of the AGE–RAGE axis holds significant potential for attenuating neuroinflammation, oxidative stress, and metabolic dysfunction, the key pathological features of AD. While no AGE-specific interventions have yet gained regulatory approval for clinical use in AD, converging lines of experimental, clinical, and epidemiological evidence underscore the translational relevance of this pathway.

Future research should prioritize the design of mechanistically informed clinical trials, the development of reliable AGE–RAGE biomarkers for early detection and stratification, and the incorporation of glycation profiles into precision medicine frameworks. Moreover, combination therapies that concurrently target glycation, mitochondrial dysfunction, and chronic inflammation may offer synergistic benefits, particularly in the early stages of LOAD.

### Open Research Questions

Despite mounting preclinical support for targeting the AGE–RAGE axis in AD, several pivotal questions remain unresolved. First, how can we systematically classify and characterize the diverse species of AGEs to enable selective therapeutic targeting? Second, what reliable and sensitive biomarkers can be developed to quantify AGE accumulation and RAGE activation in vivo, particularly within the human brain? Third, at what temporal window would AGE and AGE–RAGE-directed interventions yield maximal clinical efficacy, especially considering the likely preclinical onset of AGE-related pathology? Fourth, can emerging modalities such as nanotechnology, gene editing, or multi-target drug platforms achieve sufficient specificity, brain penetration, and safety for long-term intervention? Lastly, how can glycation-related mechanisms be effectively integrated into broader precision medicine strategies for Alzheimer’s disease, particularly in the heterogeneous context of late-onset forms? Addressing these questions will be critical for translating promising molecular insights into tangible clinical applications.

## Figures and Tables

**Table 1:** Comparison of early-onset and late-onset Alzheimer’s disease.

Feature	Early-Onset AD (EOAD)	Late-Onset AD (LOAD)
Age of onset	<65 years	≥65 years
Genetic associations	*APP*, *PSEN1*, *PSEN2* mutations	*APOE* ε4 allele, polygenic risk loci
Pathogenesis	Dominant amyloidogenic pathway	Multifactorial (metabolic, vascular, oxidative)
Oxidative stress involvement	Less prominent	Highly implicated
Frequency	~5–10% of AD cases	~90–95% of AD cases

EOAD—early-onset Alzheimer’s disease; LOAD—late-onset Alzheimer’s disease; APP—amyloid precursor protein; PSEN1—presenilin 1; PSEN2—presenilin 2; APOE—apolipoprotein E. Adapted from Seath et al., 2024 [6].

**Table 2:** Cellular biomolecular targets and effects of free radicals lead to pathological conditions.

Component	Effects of Free Radicals
Proteins	- Changes in amino acids - Peptide chain breakage - Protein denaturation - Loss of activity
Lipids	- Lipid peroxidation - Alteration of membranes
Sugars	- Glucose autoxidation
DNA	- DNA breakage - Base mutations - Alteration of gene expression

Adapted from Sultana et al., 2024 [18].

**Table 3:** Therapeutic strategies targeting the AGE–RAGE axis in AD.

Strategy	Mechanism	Example	Status	Key References
Inhibition of AGE formation	Carbonyl scavengers, Maillard reaction blockers	Aminoguanidine, pyridoxamine	Preclinical	Voziyan 2005 [58]
AGE cross-link breakers	Disruption of protein–AGE bonds	ALT-711 (Alagebrium)	Experimental	Kass 2001 [59]
Enhancement of detoxification	Upregulation of GLO1, antioxidant boosting	Sulforaphane, NAC, GSH	Investigational	Berends 2024 [60]
RAGE antagonism	Blocking AGE–RAGE interaction	Azeliragon, sRAGE	Phase II/III	Burstein 2014 [61]; Deane 2012 [62]
Lifestyle modification	Reduction of exogenous AGEs	Diet, polyphenols, exercise	Observational	Uribarri 2007 [63]; Vlassara 2002 [64]

AGE—advanced glycation end product; RAGE—receptor for advanced glycation end product; GLO1—glyoxalase 1; NAC—N-acetylcysteine; GSH—glutathione; sRAGE—soluble receptor for advanced glycation end products; NF-κB—nuclear factor kappa-light-chain enhancer of activated B cells.

## Data Availability

Not applicable.
